# Beta-nerve growth factor stimulates spontaneous electrical activity of in vitro embryonic mouse GnRH neurons through a P75 mediated-mechanism

**DOI:** 10.1038/s41598-020-67665-4

**Published:** 2020-06-30

**Authors:** Caroline Pinet-Charvet, Renaud Fleurot, Flavie Derouin-Tochon, Simon de Graaf, Xavier Druart, Guillaume Tsikis, Catherine Taragnat, Ana-Paula Teixeira-Gomes, Valérie Labas, Thierry Moreau, Xavier Cayla, Anne H Duittoz

**Affiliations:** 1 Physiologie de la Reproduction et des Comportements (PRC) UMR7247 INRA, CNRS, Centre INRA Val de Loire, Université de Tours, IFCE, 37380 Nouzilly, France; 2 Physiologie de la Reproduction et des Comportements (PRC), ComUE Centre-Val de Loire, Centre INRA Val de Loire, Université de Poitiers, 37380 Nouzilly, France; 3 Faculty of Science, School of Life and Environmental Sciences, The University of Sydney, Sydney, NSW 2006 Australia; 4 Infectiologie et Santé Publique (ISP) UMR1282, INRA, Centre INRA Val de Loire, Université de Tours, 37380 Nouzilly, France; 5 Biologie des Oiseaux et Aviculture (BOA) UMR Centre INRA Val de Loire, 37380 Nouzilly, France

**Keywords:** Reproductive biology, Neurotrophic factors, Physiology, Endocrinology

## Abstract

The control of ovulation helps guarantee the success of reproduction and as such, contributes to the fitness of a species. In mammals, two types of ovulation are observed: induced and spontaneous ovulation. Recent work on camelids, that are induced ovulators, highlighted the role of a factor present in seminal plasma, beta Nerve Growth Factor (*β*-NGF), as the factor that triggers ovulation in a GnRH dependent manner. In the present work, we characterized alpaca *β*-NGF (a*β*-NGF) and its 3D structure and compared it with human recombinant *β*-NGF (h*β*-NGF). We showed that the *β*-NGF enriched fraction of alpaca semen and the human recombinant protein, both stimulated spontaneous electrical activity of primary GnRH neurons derived from mouse embryonic olfactory placodes. This effect was dose-dependent and mediated by p75 receptor signaling. P75 receptors were found expressed in vitro by olfactory ensheathing cells (OEC) in close association with GnRH neurons and in vivo by tanycytes in close vicinity to GnRH fibers in adult mouse. Altogether, these results suggested that *β*-NGF induced ovulation through an increase in GnRH secretion provoked by a glial dependent P75 mediated mechanism.

## Introduction

Two modes of ovulation have been described in mammals: spontaneous ovulation and induced ovulation. Spontaneous ovulation occurs in many mammalian species (mouse, rat, bovine, ovine, equine, primates). In these species, ovulation occurs spontaneously whether the female copulates or not. Induced ovulation has been described in a great variety of species belonging to various mammalian Orders. Induced ovulation has been particularly well studied in camelids^1^. In these species, ovulation occurs after mating in more than 95% of females. These observations drove to the hypothesis of a physical stimulation of the female genitalia and the existence of an ovulation reflex mediated through the spine medulla. Spontaneous ovulators have been intensively studied since most laboratory species belong to this category. In these species, it is acknowledged that ovulation is triggered by an increase in luteinizing hormone (LH) levels (preovulatory surge) as the consequence of an increase in gonadotropin releasing hormone (GnRH) release. The timing of ovulation is controlled by kisspeptin neurons located in the preoptic area (POA) (ewes) or in the antero-ventral periventricular region (AVPV) (rodents)^2^. Kisspeptin stimulates GnRH secretion through an action on KISS1-R, also named GPR54 receptor, expressed by GnRH neurons^3^. In induced ovulators, ovulation is the result of an increase in LH due presumably to an increase in GnRH release, the timing of this increase is given by mating. In alpacas, intra-muscular (IM) administration of seminal plasma during the pre-ovulatory phase was able to induce ovulation in more than 90% of female alpaca^4,5^, this action being blocked by administration of a GnRH antagonist. Thus, seminal plasma contains one or several chemicals that can induce ovulation through a GnRH dependent mechanism, this factor was named Ovulation Inducing Factor (OIF)^6,7^. Early work in the bactrian camel identified two fractions of seminal plasma that were able to elicit LH secretion from in vitro culture of rat pituitary cells, suggesting the presence of a GnRH-like factor^8^. Seminal plasma was also found to be active in spontaneous ovulators such as gilts, boar ’s seminal plasma placed in gilt uterine horn advance ovulation^9^. The authors identified the activity in the 1–10 kDa fraction separated by ultrafiltration and showed that it lost its activity after treatment with pronase^9^. In 2012, two groups identified the neurotrophin beta-nerve growth factor (*β*-NGF) as the main component of OIF^10,11^.

*β*-NGF is also present in seminal plasma from spontaneously ovulating species such as bull, ram, porcine and stallion^12^. Moreover OIF activity was conserved in these species since equine and porcine seminal plasma induced ovulation in prepubertal mice^13^. Although there is strong evidence for an hypothalamic effect of *β*-NGF, other components may act at different levels such as at the pituitary level^14,15^. The purpose of our study was to determine the neuroendocrine mechanism of action of *β*-NGF using in vitro primary cultures of GnRH neurons derived from mouse embryonic nasal placode. We used *β*-NGF enriched fraction of alpaca seminal plasma (a*β*-NGF) and recombinant human *β*-NGF (h*β*-NGF). We characterized the structure of purified alpaca *β*-NGF and compared it to h*β*-NGF structure. Our study showed that recombinant h*β*-NGF and enriched fraction of alpaca seminal plasma a*β*-NGF increased GnRH neurons spontaneaous electrical activity in a dose-dependant manner. This effect was mediated by the P75 neurotrophin receptor but not by TrkA. p75 receptors were expressed by olfactory ensheathing cells in vitro and by tanycytes of the median eminence in adult mice, at the vicinity of GnRH nerve terminals suggesting a indirect effect on GnRH neurons.

## Results

### Characterization of alpaca *β*-NGF

**Figure 1:**
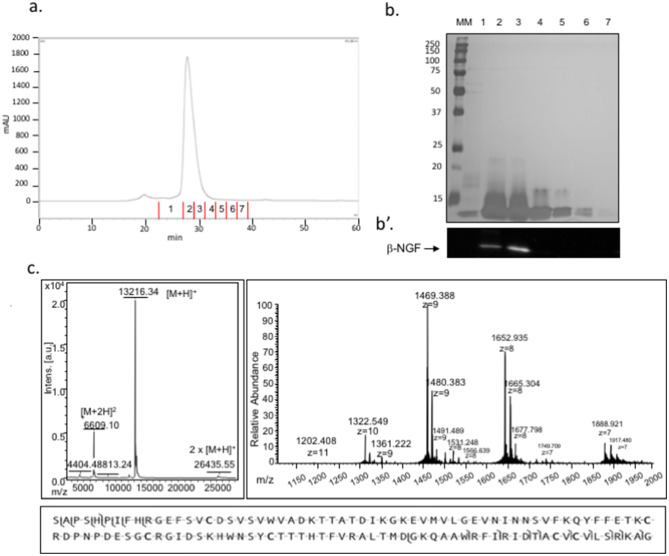
Enrichment and identification of alpaca *β*-NGF. (**a**) Representative chromatogram of seminal plasma after a Superdex 75 gel-filtration (GF) chromatography. Seven fractions were collected between 22 and 39 min of retention time. (**b**) Representative 12% SDS-PAGE profile of the 1–7 eluted GF fractions containing proteins of 13–14 kDa. Lane MM represents marker proteins with their molecular masses (kDa) indicated on the left. (**b′**) Immunodetection of the 1–7 eluted fractions using an antibody raised against human *β*-NGF. Western blotting analysis confirmed the presence of *β*-NGF and the cropped blot revealed a strong enrichment in fraction 2 and 3, at the expected molecular weight. (**c**) On the left, MALDI-TOF mass spectrum, in the mass range 4,000–30,000 m/z, of intact *β*-NGF enriched by GF chromatography. The three major peaks correspond to monocharged molecular specie [M+H]+ at 13,216.34 m/z, to bicharged molecular specie [M+2H]2+ at 6,609.10 m/z and to dimer 2 × [M+H]+ at 26435.55 m/z. On the right, multicharged ions MS spectra for intact *β*-NGF obtained with nanoESI-LTQ Velos Orbitrap. The average molecular mass of the mature *β*-NGF was observed at m/z 1,202.40; 1,322.54; 1,469.38 ; 1,652.93 and 1,888.92 respectively for the (+ 11), (+ 10), (9+), (8+) and (+7) charge states. The monocharged molecular specie [M+H]+ was calculated (mean) at $13,216.42 \pm 0.02$ Daltons. Below MS/MS fragmentation and product ion map from HCD spectra from multicharged intact *β*-NGF at m/z 1,469 and 1,652 with or without salt adducts. Fragmentation map shows the coverage of b and y fragment ions confirming N- and C-ter sequences.

Alpaca seminal plasma fraction enriched in *β*-NGF (a*β*-NGF) was obtained after fractionating seminal plasma using liquid chromatography. To further determine the sequence of alpaca *β*-NGF, we performed bottom up and top down proteomic studies. The complete sequence of mature a*β*-NGF consists of 118 amino acids displaying 97% sequence identity with llama OIF and 92% identity with human *β*-NGF (Fig. 1, see Supplementary Information Table S1).

### *β*-NGF stimulates GnRH neurons spontaneous electrical activity through a P75 mediated action

**Figure 2:**
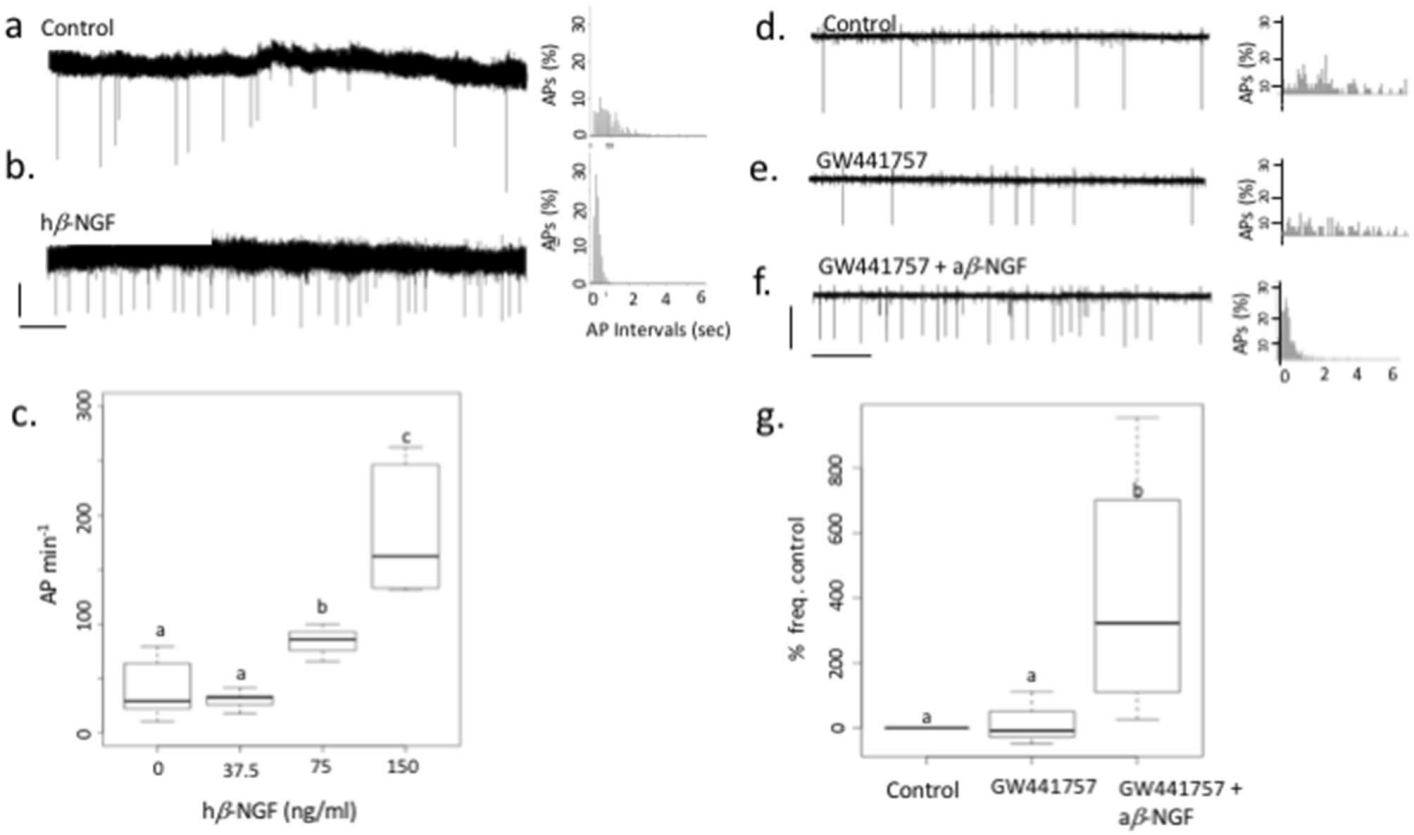
Effect of *β*-NGF on GnRH neurones spontenous electrical activity. (**a**) Spontaneous electrical activity recorded using the loose patch mode from a GnRH neuron (vertical bar = 40 pA, horizontal bar = 2 s). (**b**) Electrical activity recorded on the same neuron in (**a**) after the application of 150 ng/mL h*β*-NGF. (**c**) Boxplot representing AP frequency as a function of h*β*-NGF concentration. Different letters indicate a statistically significant difference (Kruskal–Wallis test, chi-squared = 14.588, p = 0.0022, and non parametric multiple comparisons p = 0.0098). (**d**) Control recording using the loose patch configuration of a GnRH neuron. (**e**) Trace of electrical activity recorded from the same neuron as in (**d**) after the application of 3.2 μM GW441756, TrkA antagonist. No effect on electrical activity was detected. (**f**) Electrical activity of the same neuron in (**d**,**e**). after application of $450 \, {\hbox {ng/mL a}} \beta \hbox {-NGF}$ + 3.2 μM GW441756, (**g**) boxplot representing mean AP frequency. TrkA antagonist failed to block the increase in electrical activity induced by a*β*-NGF (n = 4 experiments, paired t-test, t = − 3.8992, p = 0.0299). a′,b′,d′,e′,f′ corresponding frequency histogram of interspike intervals.

To explore the neuroendocrine mechanisms involved in the ovulation inducing properties of OIF we tested the effect of *β*-NGF enriched fraction (2+3 Fig. 1) of alpaca seminal plasma (a*β*-NGF) and recombinant h*β*-NGF on spontaneous electrical activity of GnRH neurons cultures derived from mouse embryonic nasal placodes. In control condition, electrical activity occurred at a frequency of $42.38 \pm 9.04$ action potentials (AP) per minute (AP min^−1^) (Fig. 2). This frequency was not affected by h*β*-NGF 37.5 ng/mL (n = 5, $30.3 \pm 3.9$ AP min^−1^) but was increased by 100% in the presence of 75 ng/mL (n = 3, $83.91 \pm 9.94$ AP min^−1^, Kruskal–Wallis chi-squared = 14.588 p = 0.0022,and non parametric multiple comparisons p = 0.0164) and by 300% in the presence of h*β*-NGF 150 ng/mL (n = 5, $187.09 \pm 28.11$ AP min^−1^, Kruskal–Wallis chi-squared = 14.588 p = 0.0022, and non parametric multiple comparisons p = 0.0009). This effect was reversible after washout. This increase in activity was characterized by an important increase in the proportion of short intervals between action potentials. The frequency histogram of the time intervals between AP illustrates this shortening (Fig. 2). Enriched seminal plasma fraction in a*β*-NGF 450 ng/mL (protein content) produced an 78% increase in AP frequency (n = 6, $67.02 \pm 30.18$ to $98.75 \pm 28.49$ AP min^−1^, paired t-test t = 3.3048 p = 0.0213, data not shown).

Burst analysis showed that the number of APs within a burst and burst duration increased following the application of a*β*-NGF 450 ng/mL. The number of APs within a burst and burst duration increased not immediately after a*β*-NGF 450 ng/mL application but the effect took approximately 20 min to develop. The mean intraburst interval between APs was not affected. 3. To determine which receptor was involved in this action, we first used a specific TrkA antagonist, GW441756, at 3.2 μM (IC50 = 2nM). The application of GW441756 prior or during the application of a*β*-NGF (450 ng/mL) did not prevent the increase in AP frequency (control period: $57.37 \pm 42.23$ AP min^−1^, GW441756 period: $48.53 \pm 23.76$ AP min^−1^, GW441756 + a*β*-NGF period: $124.22 \pm 38.60$ AP min^−1^, paired t-test t = − 3.899, p = 0.0299) (Fig. 2).

**Figure 3:**
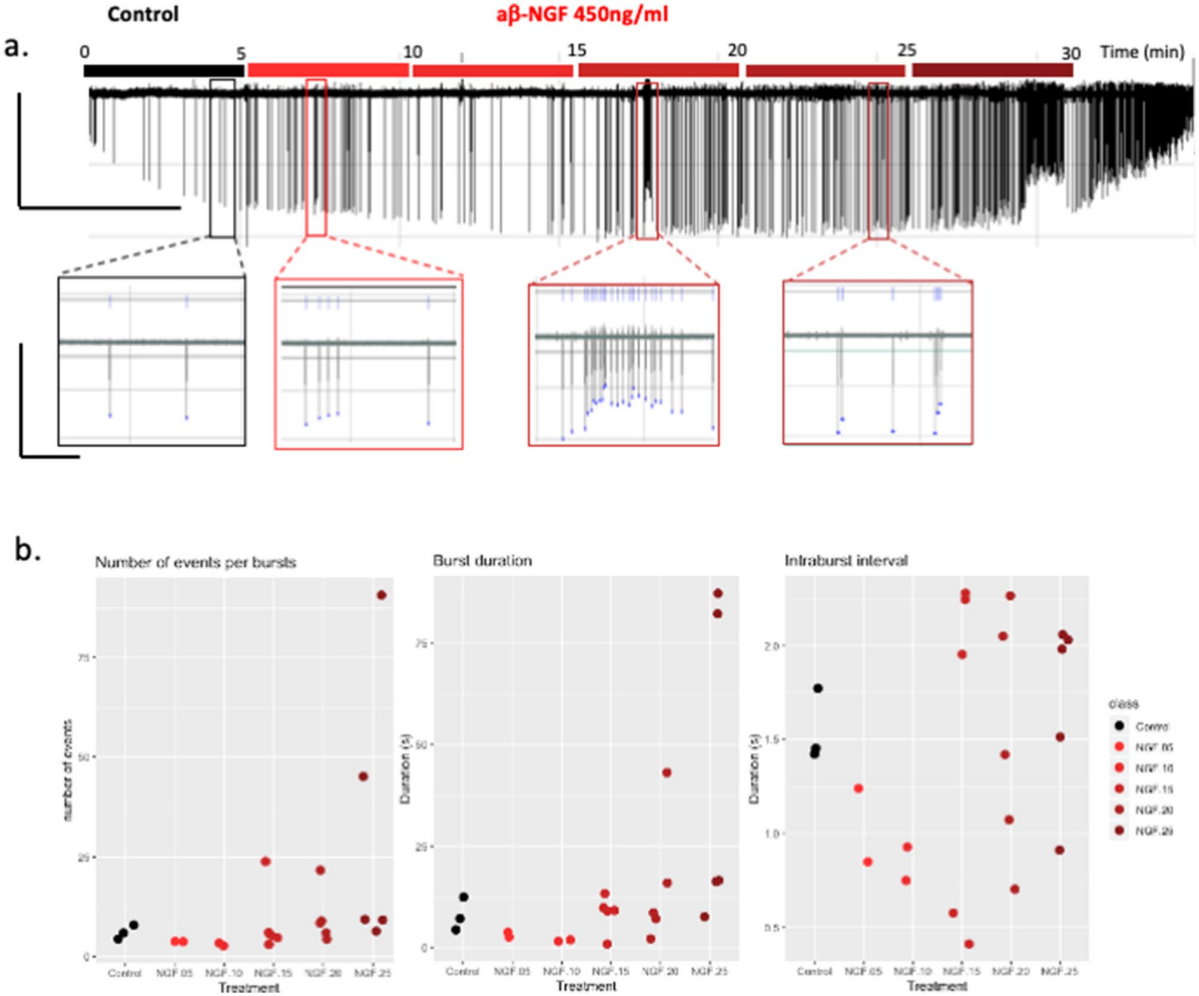
Burst analysis. (**a**) Trace recording representing a 30 min long record (horizontal bar = 5 min, vertical bar = 40 pA) and below enlarged view of typical APs or bursts of APs (horizontal bar = 12 s, vertical bar = 40 pA). The recording was divided in 5 min blocks (Control = first 5 min control condition, NGF.05 = 5 to 10 min of recording which represent 0–5 min in the presence of a*β*-NGF, NGF.10 = 5–10 min after application of a*β*-NGF, NGF.15 = 10–15min, NGF.20 = 15–20 min, NGF.25 = 20–25 min) with different color codes. (**b**) Stripcharts representing the mean number of APs within a burst, burst duration and mean intraburst interval. Following bath application of 450 ng/mL a*β*-NGF, the frequency of APs progressively increased, the number of events within a burst and the duration of burst increased. The number of bursts was 3, 2, 2, 5, 5, 5 during Control, NGF.05, NGF.10, NGF.15, NGF.20, NGF.25 periods respectively. The number of events per burst were $6 \pm 1.15$, $4.0 \pm 0.0$, $3.0 \pm 0.0$, $8.6 \pm 3.9, 9.8 \pm 3.2$, $32.0 \pm 16.4$ during control, NGF.05, NGF.10, NGF.15, NGF.20 and NGF.25 periods respectively. The mean burst duration (s) was $7.9 \pm 2.4$, $3.1 \pm 0.6$, $1.7 \pm 0.2$, $8.4 \pm 2.0$, $15.3 \pm 7.3$, $41.9 \pm 17.5$ for control, NGF.05, NGF.10, NGF.15, NGF.20 and NGF.25 periods respectively. The mean intraburst interval between APs (s) was: $1.5 \pm 0.1$, $1.0 \pm 0.2$, $0.8 \pm 0.1$, $1.5 \pm 0.4$, $1.5 \pm 0.3$, $1.7 \pm 0.2$ during control, NGF.05, NGF.10, NGF.15, NGF.20 and NGF.25 periods respectively.

**Figure 4:**
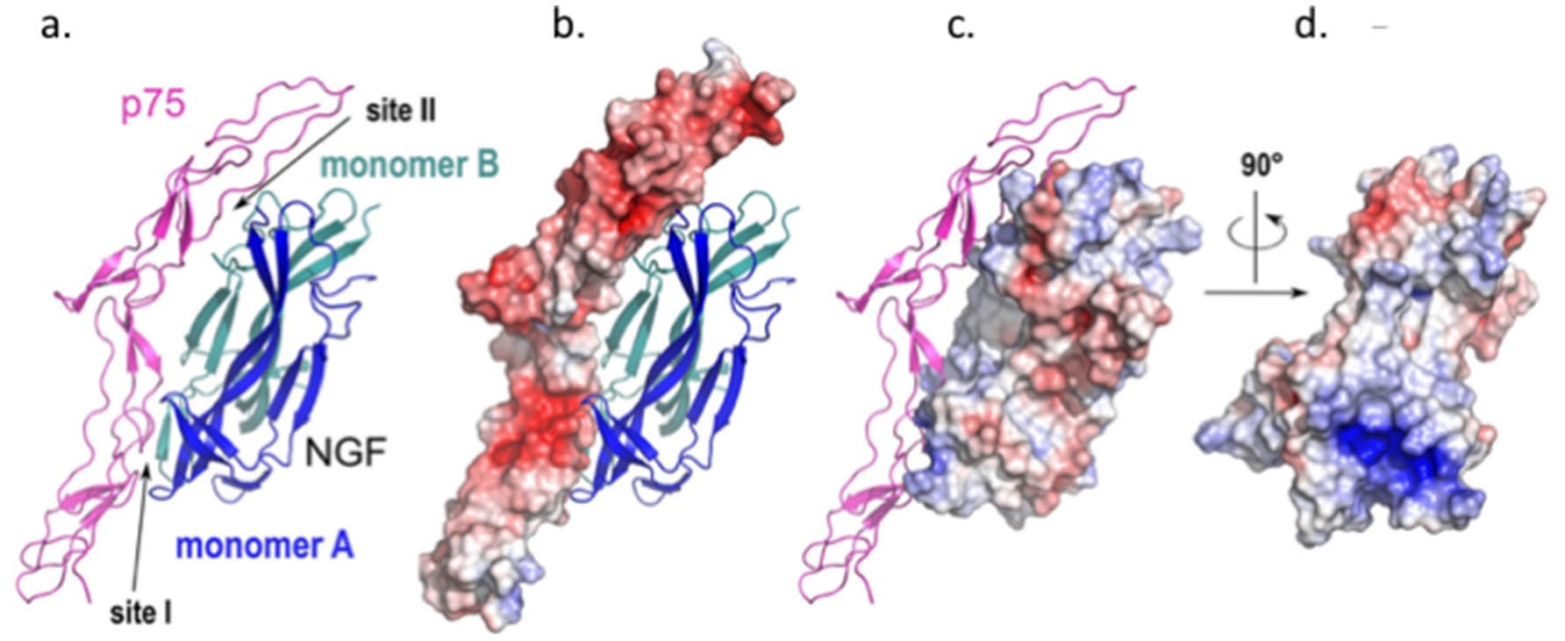
Structure of a*β*-NGF complexed with p75. (**a**) Cartoon representation of the a*β*-NGF-p75 complex where p75 (magenta) binds alongside of the alpaca homodimer model (monomer A and monomer B colored blue and cyan respectively). The solvent-accessible surface of p75 and a*β*-NGF homodimer is shown in (**b**,**c**). respectively and colored according to values of electrostatic potentials (blue:positive charges; red: negative charges). (**d**) a*β*-NGF has been rotated 90° around Y-axis to show the conserved cluster of positive charges in a*β*-NGF interaction site I. Atomic coordinates of a*β*-NGF were obtained by comparative modeling based on lama *β*-NGF using Swiss-Model server as described in Supplementary Information section. The figure was prepared with PYMOL software.

Since no p75 specific antagonist was available, we then tested a p75 receptor agonist, LM11A31 at 10 nM (EC50 = 100 nM^16^) to see whether it could mimic the effect of a*β*-NGF on GnRH neurons ’electrical activity. LM11A31 increased AP frequency from $53.36 \pm 7.87$ AP min^−1^ in control condition up to $87.07 \pm 6.22$ AP min^−1^ (n = 6, paired t-test t = -3.3908, p-value = 0.01944, data not shown). To assess the interaction of a*β*-NGF with neurotrophin receptor p75, a*β*-NGF was superimposed onto the human *β*-NGF moiety of human *β*-NGF-p75 complex (PDB 1SG1 entry code). Sixteen residues of site I and site II of a*β*-NGF are involved in interactions with 25 residues of p75, as similarly observed in p75-h*β*-NGF complex (18 a*β*-NGF residues interacting with 21 p75 residues )^17^. The buried area upon complex formation with p75 was slightly higher in the case of a*β*-NGF-p75 complex ($1318 \, {\AA }^{2}$) compared to h*β*-NGF-p75 complex ($1248 \, {\AA }^{2}$). In addition, the cluster of basic residues at interaction site I is totally conserved, providing charge complementarity between this positively charged region of a*β*-NGF and the corresponding negative region of p75 in this zone of interaction (Fig. 4).

### h*β*-NGF increased intracellular calcium peak frequency

**Figure 5:**
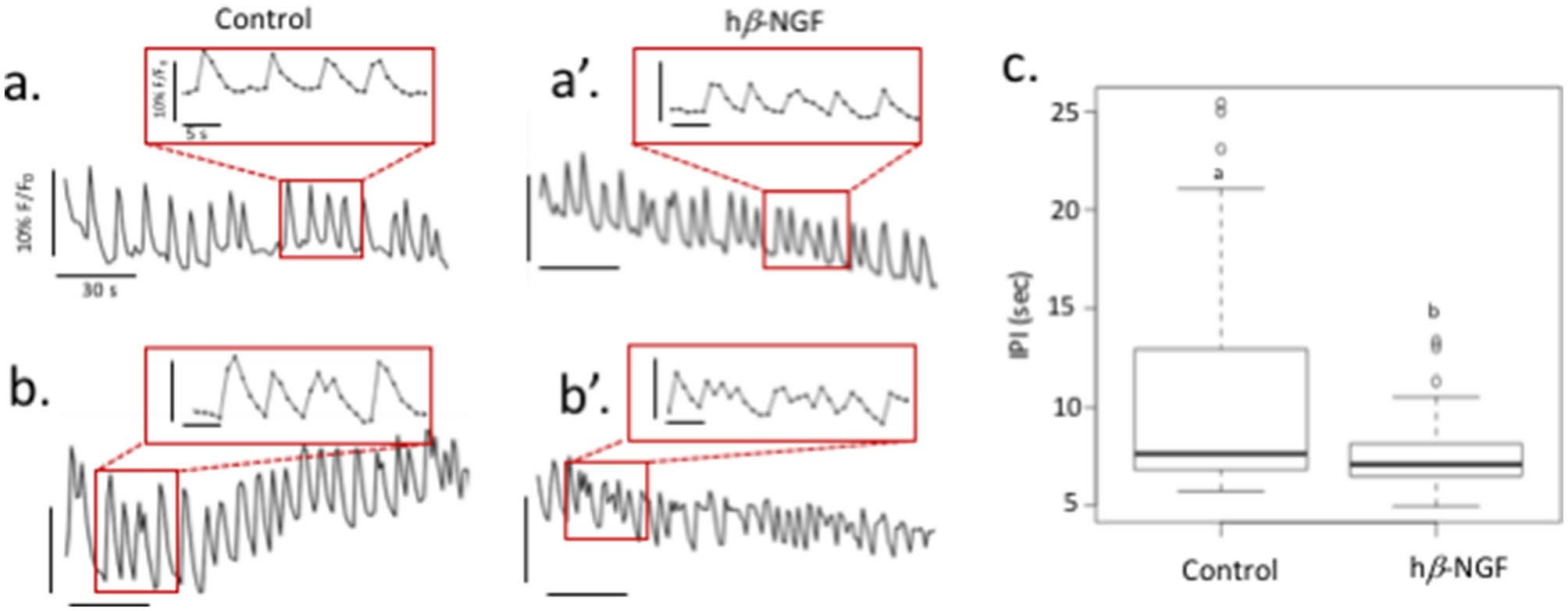
Effect of h*β*-NGF on [Ca^2+^]_I_ in GnRH neurones. (**a**,**b**) Traces of [Ca^2+^]_I_ fluctuations in individual GnRH neurons during control condition. Boxes correspond to enlarged views of traces. a’ and b’ traces recorded from same neurons after application of 75 ng/mL h*β*-NGF showing either an increase in [Ca^2+^]_I_ frequency (**a**) and an increase in [Ca^2+^]_I_ duration (**b**). (**c**) Boxplot of IPI values in control and h*β*-NGF conditions. Different letters indicate a statistically significant difference (Wilcoxon rank sum test W = 4,076, p-value = 0.0008944, n = 79)

GnRH pulsatile secretion not only depends on electrical activity but is directly related to periodic intracellular increases in [Ca^2+^]_I_. In mouse embryonic nasal explant cultures GnRH neurons present spontaneous [Ca^2+^]_I_ elevations lasting a few seconds (3–10) and occurring at a rate of 3–6 per minute. We named these events: [Ca^2+^]_I_ peaks. h*β*-NGF 75 ng/mL induced a decrease in the mean duration of [Ca^2+^]_I_ interpeaks intervals (IPI) (Fig. 5). During the control period the mean IPI was $10.42 \pm 0.58$ s (n = 79). After h*β*-NGF application the mean IPI decreased significantly to $7.47 \pm 0.17$ s (Wilcoxon rank sum test, W = 2,165, p = 0.00089). This effect on IPI was either due to an increase in [Ca^2+^]_I_ peak frequency and/or to an increase in [Ca^2+^]_I_ peak duration. We observed both mechanisms within the same experiment.

### p75 but not TrkA receptors are expressed in mouse embryonic nasal placode cultures

**Figure 6:**
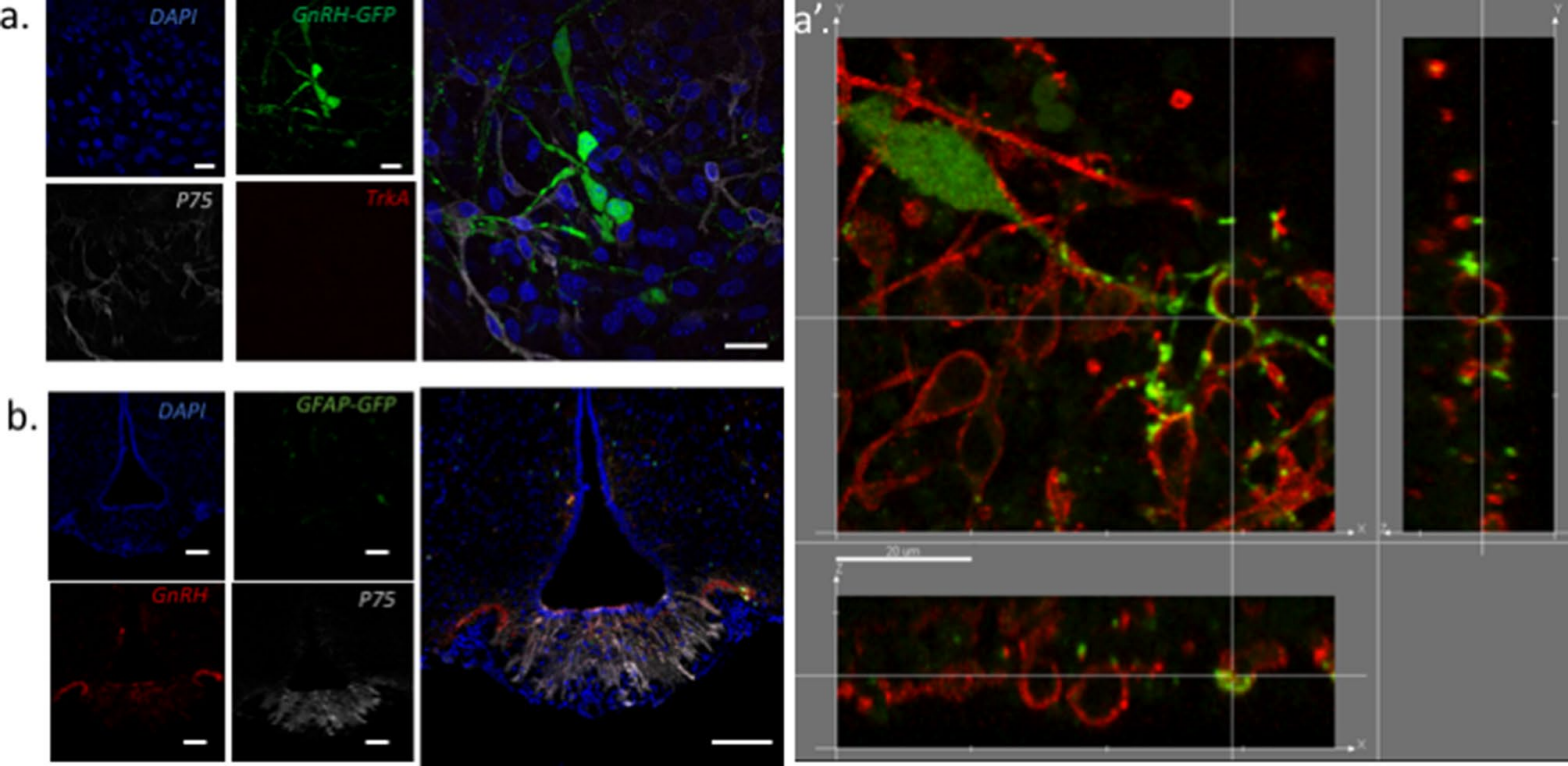
p75 immunodetection in vitro and in vivo. (**a**) In vitro immunohistochemistry. DAPI Nuclear staining. GnRH-GFP staining shows GnRH neurones ’cell bodies and neurites. p75 staining shows membrane labelled cells soma and processes, GnRH neurones were not labelled. No TrkA staining was detected (bar = 10 μm). (**a′**) In vitro immunohistochemistry of GnRH neuron was revealed with a anti-GFP (Alexa 488) here in green staining stroma and dendrites/axons. p75 was labelled with an anti P75 (Alexa 633) here in red false color. Orthographic projection shows the vicinity of GnRH neuron fibers with membranes of p75 labelled cells (bar = 20 μm). (**b**) In vivo immunohistochemistry: microphotographs of single confocal plane of hypothalamic section of a hGFAP-GFP female mouse at the level of bregma 2.06 according to the Franklin and Paxinos Mouse Brain Stereotaxic Coordinates atlas. DAPI staining . GFAP-GFP staining labelled dispersed glial cells close to the third ventricle and within the ME. GnRH staining labelled GnRH fibers in the lateral and medial external ME. P75 staining labelled tanycytes in the ME (bar = 20 μm).

p75 receptors but not TrkA receptors were detected using immunohistochemistry in GnRH neuron cultures derived from mouse embryonic nasal placodes. p75 labeling was found absent on GnRH neurons, whereas bipolar cells closely associated along to GnRH neurites and known as olfactory ensheathing cells (OEC) displayed a strong membrane labeling for P75 (Fig. 6a). Higher magnification revealed that GnRH neurites are tightly associated with p75 membrane labelled OEC, some GnRH neurites wrap around p75 membrane labelled OEC, forming some ring-like structures around GnRH-labelled structures (Fig. 6a’).

### p75 receptors are expressed in mouse median eminence

Immunodetection of p75 receptor was performed on three hypothalamic sections from three adult GFAP-GFP female mice. Figure 6b shows that neither GFAP-GFP positive cells or GnRH-labeled fibers located in the ME were labeled by p75 antibody. However, we observed a strong membrane labeling of *β*2-tanycytes in the vicinity of GnRH axons terminals (Fig. 6b).

### h*β*-NGF induced neuro-glial plasticity

**Figure 7:**
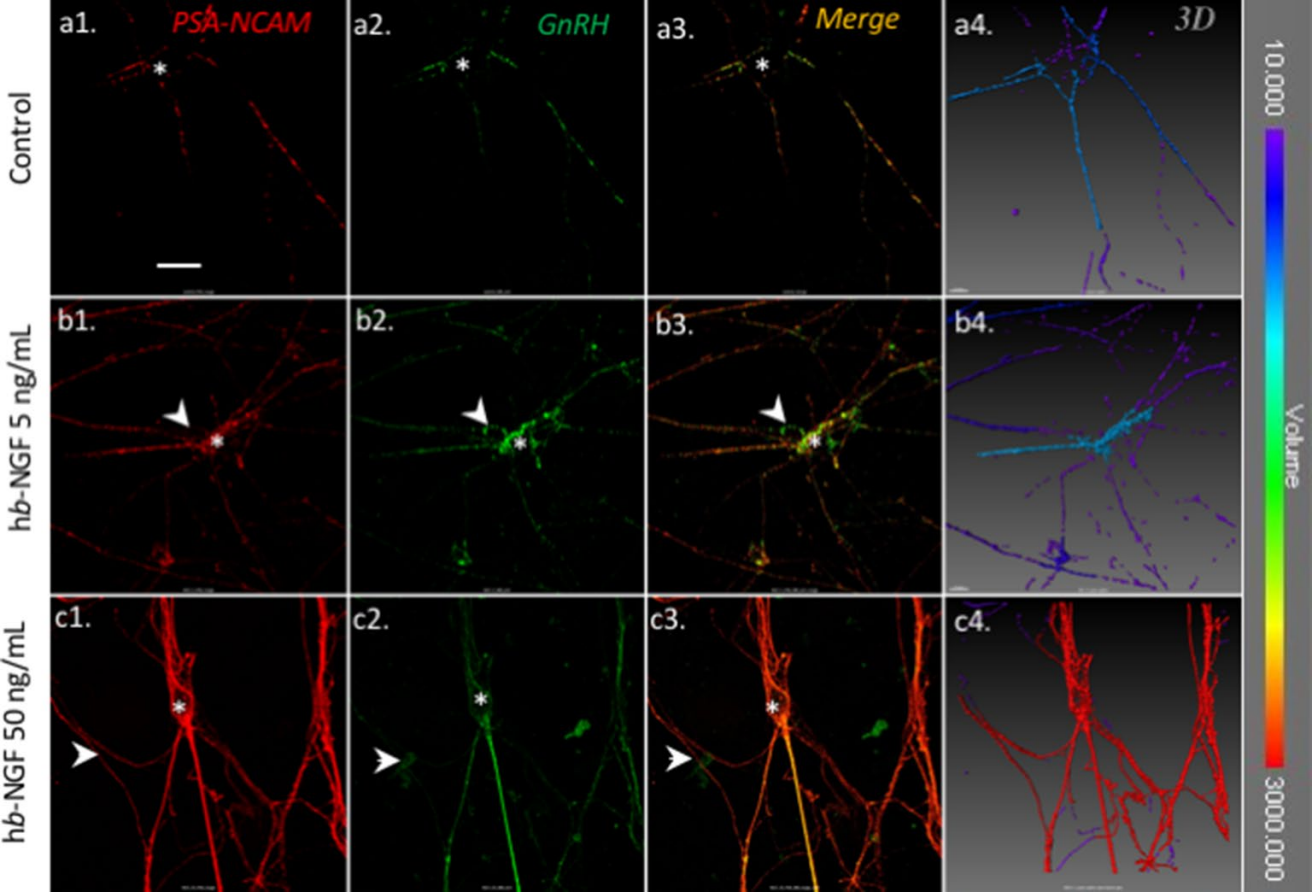
Effect of h*β*-NGF on PSA-NCAM immunoreactivity. (**a**) Control condition. (**b**) After 1 h treatment with 5 ng/mL h*β*-NGF. (**c**) After 1 h treatment with 50 ng/mL h*β*-NGF. (**a1**,**b1**,**c1**) PSA-NCAM immunoreactivity. (**a2**,**b2**,**c2**) GnRH immunoreactivity. (**a3**,**b3**,**c3**) are merge images. (**a4**,**b4**,**c4**) are 3D reconstructed images using Imaris software. False color indicate the average volume of labelled objects (see color scale from 10 to 3,000 μm).

Thus, the stimulatory effect of *β*-NGF on GnRH neurons electrical activity should be the result of p75 signaling in glial cells forming the microenvironment of GnRH neurons. To detect a possible neuroglial plasticity, we assessed polysialic acid N-Cell Adhesion Molecule (PSA-NCAM) immunoreactivity, in control or h*β*-NGF treated cultures derived from mouse embryonic nasal placodes. h*β*-NGF was applied for one hour at 5 ng/mL, a concentration without effect on APs frequency and 50 ng/mL, a concentration that was effective on electrical activity. In all conditions, we found a strong membrane labeling of GnRH neurons with PSA-NCAM antibody (stars, Fig. 7) even though some GnRH somas and fibers were not labelled by PSA-NCAM antibody (arrow’s head, Fig. 7). In control and h*β*-NGF 5 ng/mL conditions, we observed a classical patchy PSA-NCAM labeling on GnRH neuron membrane. However, after exposure to h*β*-NGF 50 ng/mL, PSA-NCAM labeling defined larger membrane patches. We assessed quantitatively these observations by measuring the volume of PSA-NCAM labelled membrane in 3D reconstructed images. In control and 5 ng/mL h*β*-NGF conditions, 100% and $91.6 \pm 7.4 \, \%$ of the total PSA-NCAM immunoreactivity volume were represented by patches smaller than 3,000 μm^3^. In contrast, patches with volumes greater than 3,000 μm^3^ formed $76.8 \pm 4.3$% of the total PSA-NCAM immunoreactive volume in 50 ng/mL h*β*-NGF treated cultures (Kruskal—Wallis rank sum test on percentage of total PSA volume arcsin transformation of class 3,000–6,000 (two classes < 3,000 and 3,000–6,000) chi-squared = 8.2927, p-value = 0.01582, Fig. 7).

## Discussion

*β*-NGF has been identified as the OIF in llama’s seminal plasma. The purified llama *β*-NGF was shown to induce ovulation in female llamas after intra-muscular (IM) or intra-vaginal administration^4^. Most in vivo studies used seminal plasma or *β*-NGF enriched seminal plasma for administration to female mammals (alpaca^4,11^, mouse^13^, cow^18^). Only one study demonstrated that purified llama *β*-NGF administrated IM to female llama induced ovulation. Since other factors with a potential effect on pituitary cells have been previously detected in seminal plasma, it is important to compare enriched *β*-NGF fraction of seminal plasma effects to recombinant *β*-NGF effects. In the present study we aimed to investigate whether *β*-NGF, either purified from seminal plasma or recombinant form, could act directly on GnRH neurons using an in vitro primary culture of GnRH neurons derived from mouse E11.5 embryo nasal placodes.

We first characterized the mature structure of alpaca *β*-NGF using top down and bottom up mass spectrometry and found 97% sequence identity with lama OIF and 92% sequence identity with human *β*-NGF.

Both alpaca seminal plasma enriched in *β*-NGF and recombinant h*β*-NGF induced an increase in spontaneous electrical activity of mouse GnRH neurones in vitro. Spontaneous electrical activity is driven by action potentials (APs). Neuronal APs involve a depolarizing step mostly due to inward sodium current provoked by the opening of voltage-gated sodium channels, this step last less than a 1 ms, followed by a repolarizing step due to potassium outward current provoked by the opening of delayed voltage potassium channels and the closure of sodium channels that are becoming refractory to depolarization. Neurons can express a variety of high voltage activated calcium channels (Cav1 (L type), Cav2 (N, P/Q and R types) which will open during the depolarizing step induced by sodium channels. The contribution of calcium current to the depolarizing phase of AP is small since calcium channels are expressed at low density compared to sodium channels. A third group of calcium channels (Cav3 or T type) are found to be expressed in neurons presenting regular phasic activity like GnRH neurons^19^, these channels are activated by low voltage and can contribute significantly to the first depolarizing step of the AP. Altogether, these channels give rise to calcium entry during the first phase of AP whose duration is less than 1 ms. Once Ca^2+^ ions reach the submembrane region of the cytoplasm, they are buffered by calcium binding proteins (calbindin, calmodulin...^20^) or by surrounding negatively charged proteins. Calcium ions can also be transported outside by the Na^+^/Ca^2+^ exchanger. These mechanisms drive Ca^2+^ ions out in a few microseconds and preclude calcium ions to diffuse outside a several 100 nm radius microdomain located around the channel inner mouth^21^. At the synapse, calcium channels are clustered and form microdomains where neurotransmitter vesicles are docked and ready for release. For neuroendocrine cells, calcium channels and large dense core vesicles (LDCV) are not located in specialized regions, such as the synapse, they are spaced at larger intervals. In neuroendocrine cells, a single AP will allow only a small number of LDCVs to be released, only the one that are located within the nanodomain of a calcium channel inner mouth will be able to fuse their membrane and release their content^22^. In contrast, a burst of AP will maintain calcium entry and [Ca^2+^]_I_ will build up, diffuse and recruit more LDVC.

Spontaneous electrical activity of GnRH neurons is carried by bursts of action potentials whose frequency determines the rhythmicity of GnRH pulsatile secretion^23^. In contrast to other neuroendocrine neurons, GnRH neuron bursts contain only two or three APs^23,24^. There are also differences according to the biological model used, bursting is observed in more than 75% of mouse hypothalamic GnRH cell line GT1–7 cells and in 75% of neurones recorded from anterior hypothalamic area (AHA) slices^25^ although this proportion varied depending on slice ’s thickness and orientation^26^. Using a ventral pharyngeal approach in anesthetized GnRH-GFP mice, Constantin et al.^27^ found a majority of neurons to have an irregular activity and only 15% were bursting. According to Lee et al.^28^, who defined a burst as “two or more spikes clustered in a 4 s interspikes intervals”, GnRH neurons derived from embryonic nasal placodes displayed bursts among irregular spiking, as described in AHA slices^23^. In the presence of h*β*-NGF, the median value of interspike interval was decreased in a dose-dependent manner and revealed an increase in the proportion of bursts. Bursts are important for neuroendocrine secretion since they induce a sufficient long lasting rise in intracellular calcium to allow hormone-containing LDCVs to be released (see^23^ for review).

Bursting electrical activity gives rise to [Ca^2+^]_I_ peaks lasting several seconds that allow GnRH local release. Partial synchronization of [Ca^2+^]_I_ transients between more than 30% of GnRH neurons was shown to be correlated with in vitro episodic GnRH release^29^. Here we showed that the inter [Ca^2+^]_I_ peak interval (IPI) was significantly decreased after application of h*β*-NGF. This decrease was either due to an increase in [Ca^2+^]_I_ peak frequency or/and to an increase in [Ca^2+^]_I_ peak duration. Both effects could be the result of an increase in the frequency and/or duration of bursts. Therefore, h*β*-NGF induced an increase in electrical activity by increasing APs frequency and by changing APs temporal distribution in favor of a bursting pattern. These changes were associated to an increase in [Ca^2+^]_I_ peaks frequency and/or duration and an increase in synchronization that could led to an increase in the probability of GnRH LDCVs release.

*β*-NGF plays multiple roles in neuronal development, function, survival, and neurite growth through an action on two types of membrane receptors: TrkA and p75^30^. Taken independently, p75 and TrkA neurotrophin receptors binds *β*-NGF with low affinity, but when they are both expressed in the same cell, p75 and TrkA forms a high affinity complex^31^. It is now well established that p75 signaling in the absence of TrkA can lead to apoptosis, cell survival, myelination or neurite outgrowth according to the partner proteins recruited in different cellular contexts^32,33^. Here we showed that the specific TrkA antagonist, GW441756, used at a concentration ten times greater than its IC50, failed to block the stimulatory effect of 450 ng/mL a*β*-NGF on GnRH neurones electrical activity. Moreover, the specific p75 agonist, LM11A31^16^, mimicked the effect of a*β*-NGF on GnRH neuron activity even at a concentration 10 times lower than its EC50. Thus, in our in vitro model, *β*-NGF seemed to increase GnRH neuron electrical activity through a p75-mediated mechanism. In embryonic nasal placode explant cultures, we did not observed p75 labelling on GnRH neurons in contrast to^34^. However, olfactory ensheathing cells (OECs), that form the cellular microenvironment of GnRH neurones in vitro, strongly expressed p75^34–37^. OECs are glial cells originating from neural crest, they ensheath GnRH neurons at their exit from the medial olfactory pit. OECs are highly plastic, they can display various glial markers and they present myelinating-like properties. OECs play a role in the nasal migration of GnRH neurons during embryogenesis^38^. OEC are tightly associated with GnRH neurons as illustrated in Fig. 7a′. Thus, at low magnification, p75 OECs’s membrane labeling might appear as belonging to GnRH neuron membrane. Similarly, the use of secondary antibodies coupled to probes whose fluorescence spectra are close may induce a fluorescence leak and an apparent membrane labelling of GnRH neurons. To prevent spectra overlap we used alexa 488- and alexa 633-coupled secondary antibodies to label anti-GnRH and anti-p75 primary antibodies, their respective spectra being sufficiently separated to avoid any overlap. Nevertheless, Raucci et al.^34^ confirmed the expression of p75 using single cell RT-PCR on isolated GnRH neurons in vitro. Thus we cannot totally exclude the fact that p75 receptor could be expressed at low level in GnRH neurons. However the strong expression exhibited by surrounding OECs, suggests that most of effects produced by *β*-NGF should be mediated through OECs p75 receptors. OECs are known to be confined in the olfactory region at adulthood, however Geller et al.^37^ demonstrated that brain lipid binding protein (BLBP)-labelled OEC progenitors accompanied GnRH neurons along peripherin fibers into the diencephalon of E15.5 mouse embryos.

Other mechanims can be involved, for example in llama, Carrasco et al.^39^, identified neurons expressing p75 and TrkA receptors in female llama’s hypothalamus. Most hypothalamic areas contained TrkA immunoreactive cells with a greater labelling in the diagonal band of Broca. P75 labelled neurons were found in the diagonal band of Broca, the lateral preoptic area and caudal parts of the hypothalamus. P75 was also detected in ependymal cells boarding the lateral ventricle and p75 fibers were observed in the *organum vasculorum of the lamina terminalis* (OVLT), and the ventral part of arcuate nucleus (ArcN) and the median eminence (ME). GnRH neurons were not labelled by p75 antibody and only 2.5 % were labelled by TrkA antibody suggesting that they are not a direct target for *β*-NGF. TrkA or p75 expressing neurons could act as a relay for *β*-NGF action on GnRH neurons^39^. The neuroanatomical location of TrkA and p75 neurons close to the third ventricle suggests that they could be sensitive to *β*-NGF in the cerebrospinal fluid. Although there is some evidence that *β*-NGF can cross the blood brain barrier^40^, the presence of TrkA and p75 neurons in the preoptic area and the hypothalamus is not a proof of interaction between TrkA/p75 expressing neurons and GnRH neurons. However, the presence of p75 immunoreactive fibers in OVLT and in the ArcN and ME, regions located outside the brain blood barrier, is of particular interest since circulating *β*-NGF could reach the dense network of GnRH fibers in these areas. We particularly focused on the ME area since it is the structure where GnRH secretion take place and can be regulated by tanycytes (see Sharif et al.^41^ for a review) and by kisspeptin terminals found in the internal layer^42^.

We found a specific p75 expression in adult mouse tanycytes of the ME, in close vicinity to GnRH fibers as previously reported in rat^43^. Tanycytes *β*1 and *β*2 form the tanycyte population localized within the median eminence. They are considered as stem cells and are involved in numerous neuroendocrine regulations and play a key role in the control of metabolism and seasonal functions^44,45^. Tanycytes are known to regulate GnRH neuronal activity through a mechanism involving the release of prostaglandin E2 (PGE2)^41,46^. They also display a cellular plasticity across the estrus cycles in female mammals^47^. To assess this cellular plasticity, we quantified PSA-NCAM labelling on GnRH neurons. The large negatively charged PSA chain of NCAM prevents homophilic interaction between cells. We used an antibody specific to the PSA group^48^, highlighting membrane regions on GnRH neurons free of adhesion with surrounding cells. h*β*-NGF treatment induced the apparition of enlarged regions labeled by PSA, suggesting a remodeling of cell-to-cell interactions. We have previously shown that PSA-NCAM is present on both OECs and GnRH neurons in vitro^49^. A previous study showed that antagonizing p75 signaling by incubation with a p75 antibody induced the defasciculation of olfactory fibers in vitro, but also decreased the surface of GnRH neurons ’soma^34^. Rearranging the glial microenvironment of GnRH neurones may lead to changes in spontaneous electrical activity as it has been described with the tripartite synapse^50–53^. In astrocytes, several mechanisms are involved in the regulation of neuronal synaptic transmission. Among them are glutamate transporters (GLT1), potassium channels, hemichannels that can regulate the synaptic strength and shape the synaptic response by controlling local glutamate and K^+^ concentrations resulting from neuronal activity^53^. Astrocytes can also release gliotransmitters such as GABA, glutamate, d-serine, adenosine/ATP that will act at the post-synaptic level (see^53^ for a review). Whether these mechanisms are functional in other types of glial cells such as OEC and hypothalamic tanycytes remains to be ascertained.

Kisspeptin terminals are found in the median eminence but in the internal layer, in contrast with GnRH terminals that are located in the external layer^42^. Attempts to detect synapses between GnRH and kisspeptin terminals in the ME have failed, thus direct regulation of GnRH secretion by kisspeptin terminals in the ME is not supported by neuroanatomy studies. However, tanycytes *β* spread their cytoplasm from the third ventricle floor lining to the capillaries in ME external layer^54^ and could be the relay between Kp and GnRH terminals.

If this mechanism is really functional in a spontaneous ovulator species is not known. Previous work by^13^ showed that injection of alpaca seminal plasma to prepubertal mice induced ovulation. However alpaca seminal plasma contains other components that could trigger ovulation either by targeting the pituitary level or the ovary level. Gene networks governing these two modes of ovulation may have been selected in various mammalian taxa under different selection pressure. Interestingly, some mammalian species displayed the two modes of ovulation. For example the domestic cat (*Felix cati*)^55,56^ and wild felid species such as the lion (*Panthera leo*)^56,57^ displayed mixed oestrus cycles. Whether the *β*-NGF and the kisspeptin pathways interact is not known and should be investigated in the future, not only on rodents but also in species displaying both modes of ovulation. In conclusion, using an in vitro approach, we showed that *β*-NGF stimulated the electrical activity of GnRH neurons favoring a bursting behavior. By increasing the frequency and/or duration of [Ca^2+^]_I_ peaks, this bursting behavior should increase the probability of GnRH release that could lead to a global increase in GnRH secretion driving LH release. All these effects seem to involve a neuro-glial plasticity mediated by p75 receptor expressed by glial cells forming the microenvironment of GnRH neurons.

## Methods

### Characterization of alpaca *β*-NGF from seminal plasma

Technical details can be found in Supplementary Information (Methods). In order to enrich *β*-NGF, seminal plasma (SP) from alpaca (*Vicugna pacos*) was fractionated by liquid chromatography (LC). SP were separated from spermatozoa by centrifugation (10,000$\times$*g* for 10 min at room temperature). The supernatant was centrifuged again (10,000$\times$*g* for 10 min at room temperature) and stored at − 80 °C.

#### Gel-filtration chromatography

Protein concentration of SP was determined using the Uptima BC Assay kit (Interchim, Montluçon, France) according to manufacturer’s instructions.

#### Gel electrophoresis

SDS-PAGE was carried out according to Laemmli’s method^58^ using a Mini-Protean II electrophoresis cell (BioRad, Marnes-la-Coquette, France).

#### Western blotting

Proteins were separated by SDS-PAGE and transferred onto nitrocellulose membrane (GE Healthcare Life Sciences WHATMAN). Semidry transfer of proteins was performed over 1.15 h at 0.8 mA/cm^2^. The membrane was then incubated with rabbit polyclonal antibody directed against human *β*-NGF (1/5000, v/v, sc11358, Santa Cruz). After being washed with TBS with 0.5% Tween 20, the membrane was incubated 1 h at 30 °C with goat anti-rabbit conjugated with peroxidase (dilution 1:5000). Blot was developed using a mixture of two chemiluminescence substrates developing kit (GE Healthcare AmershamTH ECL SelectTH Western blotting detection Reagent RPN2235 and Supersignal West Pico #34087 Chemiluminescent Substrate Thermo Scientific). Images were digitized with a cooled CCD camera (ImageMaster VDS-CL, Amersham Biosciences, GE Healthcare Life Sciences).

#### Bottom-up proteomic

In order to evaluate the purity level of *β*-NGF, GF fractions, bottom-up proteomic approach was carried out using nano-liquid chromatography tandem high-resolution mass spectrometry (nanoLC-MS/MS). Proteomic experiment using bottom-up approach was performed on a dual linear ion trap Fourier Transform Mass Spectrometer LTQ Orbitrap Velos (Thermo Fisher Scientific, Bremen, Germany) coupled to an Ultimate®3000 RSLC Ultra High Pressure Liquid Chromatographer (Thermo Fisher Scientific, Bremen, Germany) controlled by Chromeleon version 6.8 SR11 software. Data were acquired using Xcalibur version 2.1 software (Thermo Fisher Scientific, San Jose, CA), in a positive data-dependent mode in the 300–1,800 m/z mass range. Resolution in the Orbitrap was set at R = 60,000. The 20 most intense peptide ions with charge states > 2 were sequentially isolated (isolation width 2 m/z, 1 microscan) and fragmented in the high-pressure linear ion trap using CID (collision induced dissociation) mode (collision energy 35%, activation time 10 ms, Qz 0.25). Dynamic exclusion was activated during 30 seconds with a repeat count of 1. The lock mass was enabled for accurate mass measurements. Proteins were identified using SEQUEST algorithm in Proteome Discoverer software (version 2.1, Thermo Fisher Scientific) against the NCBInr database with mammalia taxonomy (download January 2018). Scaffold version 4.8.4 software (Proteome Software, Portland, USA) was used to validate protein identifications. Peptide and proteins identifications were accepted if they could be established at greater than 95.0% and 99% probability as specified by the Peptide Prophet algorithm^59^ and the Protein Prophet algorithm^60^, respectively. Protein identifications were accepted if they contained at least two identified peptides. The abundance of identified proteins was estimated by calculating the emPAI (Exponentially Modified Protein Abundance Index)^61^ using Scaffold Q+ software (version 4.8.4, Proteome Software, Portland, USA).

#### Top-down proteomic

Firstly, the fraction containing *β*-NGF (RT 27–31 min) was analyzed by Matrix Assisted Laser Desorption/Ionization Time-Of-Flight Mass Spectrometry (MALDI-TOF MS) in order to measure whole mass of intact *β*-NGF and to evaluate heterogeneity between samples.

Samples were desalted spectra were acquired using a Bruker UltrafleXtreme MALDI-TOF instrument (Bruker Daltonics, Bremen, Germany) equipped with a Smartbeam laser at 2 kHz laser repetition rate following an automated method controlled by FlexControl 3.0 software (Bruker Daltonics, Bremen, Germany). Spectra were obtained in positive linear ion mode in the 1,000–20,000 m/z range. Secondly, 3 μL of *β*-NGF desalted by SPE and diluted with 40 μL of 50% (v/v) methanol/1% (v/v) FA was loaded into a metalized nanoelectrospray needle (PicoTip emitters, New Objective, USA). Top-Down MS and MS analyses were performed on a LTQ Orbitrap Velos instrument (Thermo Fisher Scientific, Bremen, Germany) operating in positive mode. MS and MS/MS data were acquired using Xcalibur version 2.1 software (Thermo Fisher Scientific, San Jose, CA).

#### 3D modeling

The alpaca *β*-NGF sequence was submitted to the automated protein structure homology-modelling server Swiss-Model (http://swissmodel.expasy.org). A 3D model of alpaca *β*-NGF was generated based on the llama OIF X-ray structure (PDB 4EFV entry code) as a template. Because alpaca *β*-NGF exhibits 97% sequence identity with llama OIF, the modeling procedure was straightforward and the corresponding root mean square (rms) between both structures was very low (0.096 Å). To assess the interaction of alpaca NGF with neurotrophin receptor p75, alpaca *β*-NGF was superimposed onto the human *β*-NGF moiety of human *β*-NGF-p75 complex (PDB 1SG1 entry code). As expected from sequence identity of 92% between both *β*-NGF, their 3D structure were perfectly superimposed (not shown). Most, if not all interactions, between p75 and each *β*-NGF were conserved, as shown by analysis of the interface of p75 with each *β*-NGF using COCOMAPS tools (https://www.molnac.unisa.it/BioTools/cocomaps/).

### Animals

hGFAP-GFP mice (Charles River), Swiss mice (Janvier Labs, Saint Genest L’isle, France ), GnRH-GFP mice (donated by Daniel Spergel^62^) were housed in the rodent facility at UEPAO, INRA centre Val de Loire, Nouzilly France. Each cage housed four mice at − 20 °C, which were used to being housed together.

### Primary culture of embryonic nasal placode explants

Pregnant GnRH-GFP mice at embryonic day 11.5 were killed by cervical dislocation. Briefly, embryos were placed in ice cold Gey’s balanced salt solution (Eurobio) enriched with 5% glucose (Sigma-Aldrich). Nasal pits were dissected out and adhered onto glass coverslips (Ø14 mm Marienfeld) coated with chicken plasma/thrombin (Sigma-Aldrich). Nasal explants were maintained in a defined serum-free medium (SFM)^29,63^.

### Electrophysiology

Nine cultures for experiments with a*β*-NGF and twenty-one cultures for experiments with h*β*-NGF, at 7–14 DIV were transferred to a recording chamber (RC-25 reference 64-0232 from Warner Instruments) under a fixed-stage microscope (Leica DM IRB 275064) in a bath solution containing (in mM) NaCl 124, KCl 4.1, CaCl_2_ (2H_2_O) 1, MgCl_2_ (6H_2_O) 0.3, MgSO_4_ (7H_2_O) 0.4, NaHCO_3_ 20.1, NaH_2_PO_4_ (2H_2_O) 1, and glucose 35; pH (NaOH) 7.4 (see^63^ for details). Loose patch recordings were performed at (37 °C) (Automatic Temperature controller TC-324B Warner Instruments Corp) and acquired using Clampex 10 (25 kHz sampling and low pass 5 kHz filtering) (Molecular Devices) connected to an Axopatch 200A patch-clamp amplifier and digitized with a Digidata 1550B A/D converter^63^. Patch electrodes (5–10 M$\Omega$)^63^ were filled with a solution containing (in mM): NaCl 120, KCl 4.7, CaCl_2_ (2H_2_O) 2.6, MgCl_2_ (6H_2_O) 2, MgSO_4_ (7H_2_O) 0.7, HEPES 10 and glucose 10; pH = 7.4 with NaOH^63^. Peak detection was performed with Clampfit. Burst analysis used the Poisson surprise method in Clampfit software.

### Calcium Imaging

Calcium imaging recordings were performed between 7 and 14 DIV (n = 79 neurons, N = 3 cultures). Explant cultures were loaded with the calcium-fluorescent dye Green-1 AM (10 μM, Thermofisher, Villebon sur Yvette, France) (see^63^ for details). Cultures were mounted in a perfusion chamber (Warner Instruments, Hamden, CT, USA) and loaded with caclium Green-1 AM solution contanining 2.5mM probenicid (blocker of organic anion transporter) and 10 mM Hepes in SFM^63^. Fluorescence was visualized using an inverted microscope (DM-IRB; Leica Microsystems GmbH) and acquired through a × 20 objective using a cooled intensified charge-coupled device camera (CoolSNAP HQ2; Ropper Instruments, Photometrics) using Metafluor software (Molecular Devices). Excitation wavelengths were provided by a xenon lamp through a YFP-2427b filter block (BrightLine Single band filter set Semrock, excitation bandpass filter 488–512 nm, dichroic edge wavelength at 520 nm, emission bandpass filter 528.5–555.5 nm, 93% transmission)^63^. Fluctuations in [Ca^2+^]_I_ were analyzed with Metafluor software. Each cell was individually identified as a circular region of interest (ROI). Excitation duration was set to 100 ms and time lapse acquisition was set to 1 Hz. Calcium Green-1 mean fluorescence intensity for each region of interest was calculated and analyzed with Excel (Microsoft Corp). A calcium peak was defined as a value greater than the average of the five previous points + 1.5 × standard deviation^63^.

### Tissue processing

hGFAP-GFP mice were anaesthetised with pentobarbital (150 mg/kg IP) and transcardially perfused with 15 mL of 4% paraformaldehyde in PBS. Brains were dissected out of the skull and post-fixed in 4% PAF for 24 h. The brains were then soaked in 20% sucrose solution (PBS with 0.1% sodium azide) at 4 °C for 24 h. Brains were then embedded in Tissu-Tek (Sakura, Finetek Europe BV, Zoeterwoude, The Netherlands) and frozen in isopentane solution at − 50 °C and stored at − 20 °C until sectioning. Coronal 20 μm sections were made using a cryostat (CM3050S Leica Microsystemes from the beginning of the hypothalamus to the mammillary bodies. Tissue sections were stored at − 20 °C until processing for immunohistochemistry.

### Immunocytochemistry

#### Cell culture p75, TrkA, GnRH-GFP immunohistochemistry

After 10 days in vitro (DIV), cultures were fixed in 4% formaldehyde (30–45 min). Nonspecific binding sites were blocked in PBS supplemented with 5% normal goat or horse serum or 2% BSA, 0.3% Triton X-100 and 0.1% sodium azide and incubated overnight at 4 °C with polyclonal goat anti-GFP (1:2000, Abcam ab5450), polyclonal rabbit anti-p75 (1:1000, promega ref G3231), monoclonal mouse anti-TrkA (1:1000,Merk Millipore ref 06-574). The next day, cultures were rinsed in PBS and incubated in secondary antibodies (anti-goat Alexa Fluor 488, 1:500 and anti-rabbit Alexa 633, 1:100, and anti-mouse Alexa 594, 1:500, for 2 h at room temperature), then rinsed and counterstained using the nuclear dye DAPI (1 mM), 1:5000, Sigma Aldrich, Illkirch, France) and mounted with Fluomount G (Sigma Aldrich, Illkirch, France).

#### Cell culture PSA-NCAM, GnRH and GFAP immunohistochemistry (IHC)

Six cultures aged between 13 DIV and 16 DIV were exposed either to control SFM or to h*β*-NGF 5 or 50 ng/mL for 1 h and fixed in 4% formaldehyde (30–45 min). Nonspecific binding sites were blocked as described above, and incubated overnight at 4 °C with monoclonal mouse IgM anti-polysyalylated neural cell adhesion molecule (PSA-NCAM) (1:5000, Abcam, ref: abc0019). The following day after PBS rinses, cell cultures were incubated in goat anti mouse IgM coupled to Rhodamine (1:500, Sigma Aldrich, ref: AP128R) for two hours at room temperature. After PBS rinses, cell cultures were then incubated with monoclonal mouse IgG anti-GnRH SMI-41 (Abcam ref: ab24563) and polyclonal IgG rabbit anti-GFAP (1:500, DAKO ref: Z0334) overnight at + 4 °C. The following day after PBS rinses, cell cultures were incubated with biotinylated goat anti mouse IgG (1:200) and goat anti rabbit IgG coupled to Alexa 642 (1:500) for 2 h at room temperature. After PBS rinses, cell cultures were incubated in streptavidine coupled to AMCA (1:500) for 30 min, and mounted with Fluomount G (Sigma Aldrich, Illkirch, France).

#### In vivo p75, GnRH, GFAP-GFP immunohitochemistry (IHC)

Nonspecific binding sites were blocked as previously described and incubated overnight at + 4 °C with polyclonal chicken anti-GFP (1:10000, Aves Lab), polyclonal rabbit anti-p75 (1:1000, promega ref G3231), polyclonal sheep anti-GnRH (1:5000, gift from Dr Alain Caraty)^37,63^. Sections were then rinsed in PBS and incubated for two hours at room temperature in secondary antibodies Alexa 488 donkey anti-chicken IgY (1:500), Alexa 633 donkey anti rabbit IgG (1:500) and Alexa 546 donkey anti sheep IgG (1:500). All secondary antibodies are from Thermofisher, Villebon sur Yvette, France). Sections were conterstained with DAPI, and mounted in Fluomount G (Sigma Aldrich, Illkirch, France).

### Fluorescence microscopy and image analysis

Stack image acquisition was performed using a laser confocal microscope (LSM700 Zeiss, Germany) and the associate Zen software. Images 1024 × 1024 pixels were acquired sequentially. At the objective Plan apochromat 40×/1.3 oil DIC M27, the x, y resolutions were 0.156 μm, 0.156 μm and the z resolution was 0.5 μm. Images were analysed using FIJI software. Volumes of of PSA-NCAM labelled structures from IHC image stacks were determined with Imaris software (release 9.2, Bitplane AG, Zurich, Switzerland). In image stacks 8 bits encoded, a threshold of intensity of 10 was used to eliminate background and pixel with intensity above 247 were eliminated. The segmented volumes quantified where above 2 μm^3^.

### Statistical analysis

All statistical analyses were performed using R Studio^64^. Two-samples comparisons were performed using Student test if the variable distribution followed a Normal law (Shapiro-Wilk test) and variances were homoscedatic (Fisher test). If these hypothesis were not verified we used non-parametric Wilcoxon ranking test). Multiple samples comparisons were performed using non parametric Kruskal–Wallis followed by post hoc non-parametric multiple comparisons (mctp function, nparcomp package). Chi-square test was used to compare the proportion of prepubertal mice having ovulated according to the treatment group. The number of COCs obtained according the treatment was analysed using a permutation multivariate analysis of variance. The comparison of average percentage of volumes for PSA-NCAM labelling between control and h$\beta$-NGF treatments was made after arcsin transformation by Kruskal–Wallis non parametric test.

### Ethics statement

All animals ’procedures were carried out with permission from the Val de Loire Ethics Committee of the French Ministry of Agriculture, in accordance with the European legislation for animal experimentation (Animal housing Authorization F371752, protocol authorisation no. 10378-2017052213185465 from the French Ministry of Agriculture). All experiments described were conducted in accord with accepted standards of humane animal care and all efforts were made to minimise suffering.

## Supplementary information

Supplementary figure S1

Supplementary information

## Data Availability

The datasets generated during and/or analysed during the current study are available from the corresponding author on reasonable request.
