# Supplementary material for: Beta-nerve growth factor stimulates spontaneous electrical activity of in vitro embryonic mouse GnRH neurons through a P75 mediated-mechanism
Source: Sci Rep. 2020 Jun 30;10:10654. doi: 10.1038/s41598-020-67665-4 (PMC7326925; doi:10.1038/s41598-020-67665-4)
Supplement: Supplementary file 1 — Supplementary figure S1 [file 41598_2020_67665_MOESM1_ESM.pptx]

## Slide 1
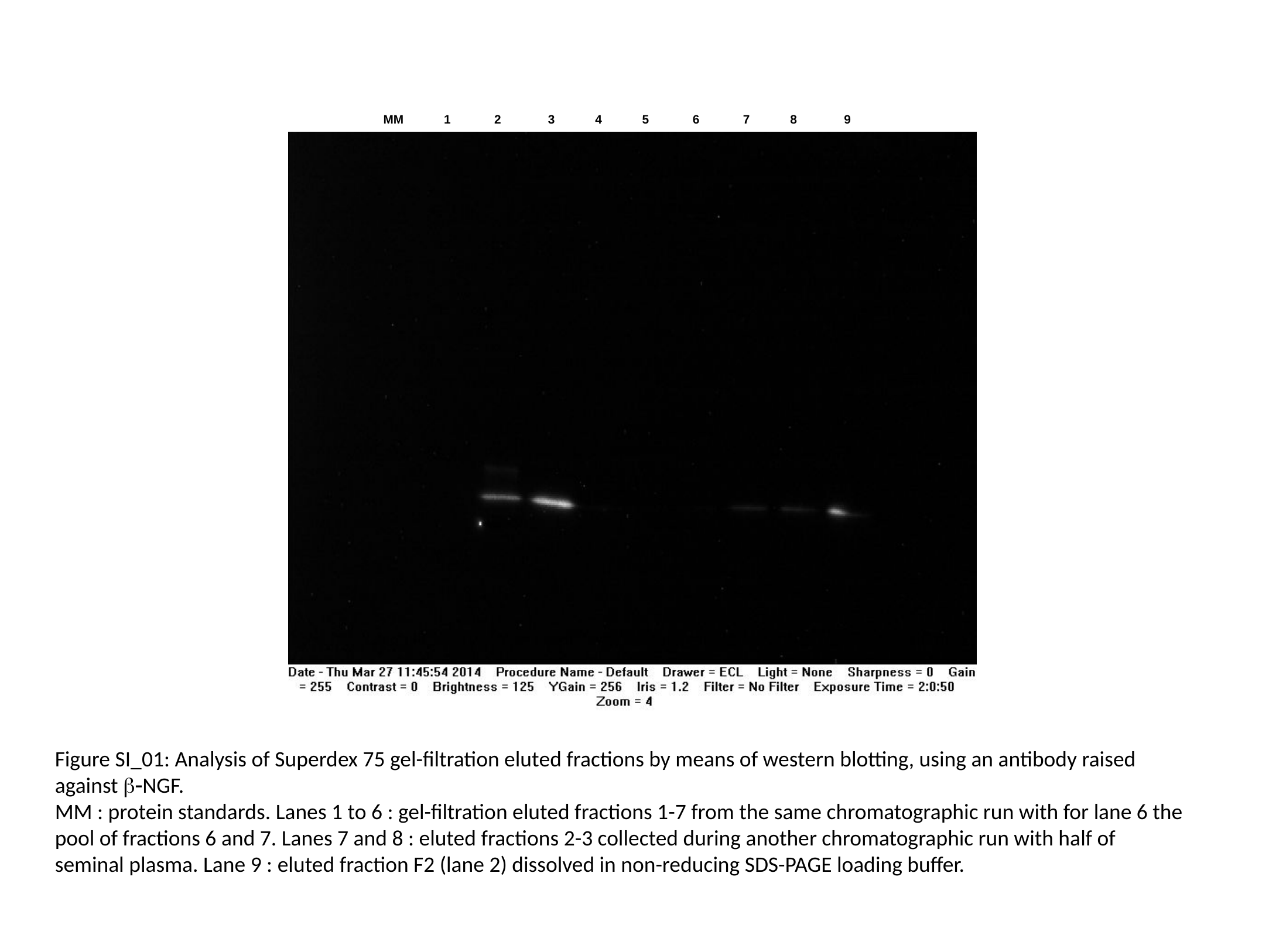

MM 1 2 3 4 5 6 7 8 9
Figure SI_01: Analysis of Superdex 75 gel-filtration eluted fractions by means of western blotting, using an antibody raised against b-NGF.
MM : protein standards. Lanes 1 to 6 : gel-filtration eluted fractions 1-7 from the same chromatographic run with for lane 6 the pool of fractions 6 and 7. Lanes 7 and 8 : eluted fractions 2-3 collected during another chromatographic run with half of seminal plasma. Lane 9 : eluted fraction F2 (lane 2) dissolved in non-reducing SDS-PAGE loading buffer.
