# Supplementary material for: Beta-nerve growth factor stimulates spontaneous electrical activity of in vitro embryonic mouse GnRH neurons through a P75 mediated-mechanism
Source: Sci Rep. 2020 Jun 30;10:10654. doi: 10.1038/s41598-020-67665-4 (PMC7326925; doi:10.1038/s41598-020-67665-4)
Supplement: Supplementary file 2 — Supplementary information [file 41598_2020_67665_MOESM2_ESM.docx]

beta-Nerve Growth Factor triggers ovulation bystimulating GnRH neurons through a P75 mediated-mechanism

Caroline Pinet-Charvet, Renaud Fleurot, Flavie Derouin-Tochon, Simon de Graaf, Xavier Druart, Guillaume Tsikis, Catherine Taragnat, Ana-Paula Teixeira-Gomez, Valerie Labas, Thierry Moreau, Xavier Cayla, and Anne H Duittoz

**Supplementary Information**

**SI Table 1**

| Accession Number | Gene ID | Protein description | Number of Unique peptides | Number of Unique peptides and sequences | Sequence coverage | emPAI |
| --- | --- | --- | --- | --- | --- | --- |
| XP_006193233.1 | NGF | PREDICTED: beta-nerve growth factor | 11 | ALTMDGKQAAWR  CRDPNPDESGCR  FIRIDTACVCVLSR  FIRIDTACVCVLSRK  GIDSKHWNSYCTTTHTFVR  HWNSYCTTTHTFVR  IDTACVCVLSR  IDTACVCVLSRK  QYFFETKCRDPNPDESGCR  SAPSHPIFHRGEFSVCDSVSVWVADKTTATDIK | 40 % | 4.7 |
| XP_021019162.1 | Alb | Serum albumin | 3 | DVFLGTFLYEYSR  GLVLIAFSQYLQK  YNDLGEQHFKGLVLIAFSQYLQK | 6 % | 0.15 |
| XP_015094906.1 | LOC  107033539 | PREDICTED: double-headed protease inhibitor, submandibular | 2 | CLFCSAVAR  VSPVPKGPTYILEEST | 26 % | 0.75 |
| XP_006184543.1 | NPC2 | PREDICTED: epididymal secretory protein E1 | 2 | DCGSVVGVIK  SGINCPIQK | 13 % | 0.43 |
| NP_000143.2 | GAA | Lysosomal alpha-glucosidase preproprotein | 2 | GGEARGELFWDDGESLEVLER  STGGILDVYIFLGPEPK | 4 % | 0.06 |

**Table 1:** Identification of proteins in the gel filtration (GF) fraction (RT 27-31 min).

After in-solution digestion by trypsin and nanoLC-MS/MS analysis, results are given by computational analysis of bottom-up proteomic data: NCBInr accession number, gene identification, protein description, number of unique peptides and corresponding sequence, sequence coverage (%) and absolute quantification using emPAI algorithm. This analysis revealed β-NGF as the major component.

**SI Material and methods**

*Gel-filtration chromatography*

For each purification run, 0.63 mg of SP total protein was loaded onto a gel filtration (GF) column (Superdex 75 10/300 GL, GE Healthcare Europe GmbH, Velizy-Villacoublay, France). GF chromatography process was carried out at room temperature, at a flow rate of 0.4 mL per minute using an Ultimate^®^ 3000 RSLC system controlled by Chromeleon version 6.8 SR13 software (Thermo Fisher Scientific, Bremen, Germany). Elution was performed isocratically using 100 mM ammonium bicarbonate buffer. UV absorption was monitored at 280 nm and fractions were collected every two minutes. Each fraction was analyzed by both western blot and by mass spectrometry using bottom-up or top-down approaches. Fractions containing β-NGF were pooled and lyophilized.

*Gel electrophoresis*

Gels were run at a constant voltage of 200 V for 1 h at room temperature including a molecular weight marker (Precision Plus Protein^TM^ All Blue Standards, Bio-Rad, Hercules, USA). Proteins were detected by silver staining.

*Western blotting*

The membrane was blocked with Tris-buffered saline (TBS) containing Tween 20 (0.5% (w/v)), supplemented with lyophilized low-fat milk (5% w/v). The membrane was then incubated with rabbit polyclonal antibody directed against human β-NGF (1/5000, v/v, sc11358, Santa Cruz) diluted in TBS-Tween containing low-fat milk (5% w/v) under mild agitation overnight at 4°C.

*Bottom-up proteomic*

The GF chromatography fraction containing β-NGF (RT 27-31 min) was digested with bovine trypsin (sequencing grade, Roche Diagnostics, Germany). Proteins were reduced by dithiothreitol 5 mM during 30 min at 56°C and alkylated by iodoacetamide 12.5 mM during 20 min at room temperature in the dark. Proteins were digested overnight with trypsin using a ratio 1:40 enzyme/substrate.

Proteomic experiment using bottom-up approach was performed on a dual linear ion trap Fourier Transform Mass Spectrometer LTQ Orbitrap Velos (Thermo Fisher Scientific, Bremen, Germany) coupled to an Ultimate\textsuperscript{®} 3000 RSLC Ultra High Pressure Liquid Chromatographer (Thermo Fisher Scientific, Bremen, Germany) controlled by Chromeleon version 6.8 SR11 software. Five µL of peptides were injected on a trap column and separated on a nano-column as previously described by Labas et al.*^1^*. Sample was desalted and concentrated for 10 min at 5 µL/min on an LCPackings trap column (Acclaim PepMap 100 C18, 75 µm inner diameter x 2 cm long, 3 µm particles, 100 Å pores). Peptides separation was conducted using a LC Packings nano-column (Acclaim PepMap C18, 75 µm inner diameter x 50 cm long, 2 µm particles, 100 Å pores) at 300 nL/min by applying gradient consisted of 4–55% B during 90 min. Mobile phases consisted of (A) 0.1% formic acid, 97.9% water, 2% acetonitrile (v/v/v) and (B) 0.1% formic acid, 15.9% water, 84% acetonitrile (v/v/v).

Data were acquired using Xcalibur version 2.1 software (Thermo Fisher Scientific, San Jose, CA), in a positive data-dependent mode in the 300-1800 m/z mass range. Resolution in the Orbitrap was set at R = 60,000. The 20 most intense peptide ions with charge states > 2 were sequentially isolated (isolation width 2 m/z, 1 microscan) and fragmented in the high-pressure linear ion trap using CID (collision induced dissociation) mode (collision energy 35%, activation time 10 ms, Qz 0.25). Dynamic exclusion was activated during 30 seconds with a repeat count of 1. The lock mass was enabled for accurate mass measurements. Polydimethylcyclosiloxane (m/z, 445.1200025, (Si(CH_3_)_2_O)_6_) ion was used for internal recalibration of the mass spectra.

Proteins were identified using SEQUEST algorithm in Proteome Discoverer software (version 2.1, Thermo Fisher Scientific) against the NCBInr database with mammalia taxonomy (download January 2018).

Database search criteria included trypsin as a protease with two missed cleavages allowed, variable modifications (carbamidomethylcysteine, methionine oxidation and acetylation of N-terminal protein). The tolerance of the ions was set to 5 ppm for parent and 0.8 Da for-fragment ion matches.

Scaffold version 4.8.4 software (Proteome Software, Portland, USA) was used to validate protein identifications. Peptide and proteins identifications were accepted if they could be established at greater than 95.0\% and 99\% probability as specified by the Peptide Prophet algorithm *^2^* and the Protein Prophet algorithm *^3^*, respectively. Protein identifications were accepted if they contained at least two identified peptides. The abundance of identified proteins was estimated by calculating the emPAI (Exponentially Modified Protein Abundance Index) *^4^* using Scaffold Q+ software (version 4.8.4, Proteome Software, Portland, USA).

*Top-Down proteomic*

Twenty µL of fraction were desalted and concentrated by solid phase extraction (SPE) using Zip Tip C4 (Millipore, Saint-Quentin-en-Yvelines, France) with 0.1% trifluoroacetic acid (TFA) and eluted with 4 µL of 50% (v/v) acetonitrile 0.1% (v/v) TFA and 4 µL of 100% acetonitrile. Samples were air-dried using SpeedVac and redissolved in 4 µL of 1% formic acid. One microliter of sample was overlayed with 1 µL of sinapinic acid matrix dissolved in 50% acetonitrile/ 50% water in presence of 0.1% TFA. The matrix/sample mix was deposed onto a polished steel 384 MALDI plate (Bruker Daltonics, Bremen, Germany) and was allowed to evaporate at room temperature.

Samples were desalted spectra were acquired using a Bruker UltrafleXtreme MALDI-TOF instrument (Bruker Daltonics, Bremen, Germany) equipped with a Smartbeam laser at 2 kHz laser repetition rate following an automated method controlled by FlexControl 3.0 software (Bruker Daltonics, Bremen, Germany). Spectra were obtained in positive linear ion mode in the 1,000–20,000 m/z range. Each spectrum was collected as a sum of 1,000 laser shots in 5 shot steps (total of 5,000 spectra).

The parameters used for spectra acquisition were: ion source 1, 25 kV; ion source 2, 22.35 kV; lens, 7.95 kV; pulsed ion extraction; and laser parameter set, medium. External calibration using cubic enhanced algorithm was followed using a mixture of peptides and proteins (1 µL of SA matrix plus 1 µL of calibrant solution containing insulin and ubiquitin, both at 1 pmol/µL, 2 pmol/µL cytochrome C, 4 pmol/µL myoglobin and 8 pmol/µL trypsinogen. Spectrum was processed (comprising Top Hat baseline subtraction, two smoothing using the Savitzky-Golay algorithm at 5 m/z width, automatic peak detection with a signal/background noise greater than 3) and visualized using flexAnalysis 4.0 software (Bruker Daltonics, Germany).

Secondly, 3µL of β-NGF desalted by SPE and diluted with 40 µL of 50\% (v/v) methanol/1\% (v/v) FA was loaded into a metalized nanoelectrospray needle (PicoTip emitters, New Objective, USA). Top-Down MS and MS analyses were performed on a LTQ Orbitrap Velos instrument (Thermo Fisher Scientific, Bremen, Germany) operating in positive mode. MS and MS/MS data were acquired using Xcalibur version 2.1 software (Thermo Fisher Scientific, San Jose, CA). Standard mass spectrometric conditions for all analyses were spray voltage 1.1-1.4 kV, no sheath and auxiliary gas flow; heated capillary temperature, 200 °C; predictive automatic gain control enabled, and an S-lens RF level of 60%. Source fragmentation energy was set at 10 V. The analysis was performed manually both the MS spectrum using the profile mode in the 400-2,000 m/z mass range and the MS/MS spectrum using HCD (High-Energy Collisional Dissociation) fragmentation mode with variable collision energy between 20-40V. Target resolution was 100,000 for MS and MS/MS analysis. The selected precursor width for fragmentation was 2-3 m/z. Spectra corresponded to the accumulation of scans over approximately 10 min, yet good signal to noise ratios could be obtained within less time. Structural characterization was performed using ProSight PC software 4.0 (Thermo Scientific, San Jose). Raw data file was processed by THRASH algorithm (signal/noise: 2) to convert each fragmentation multi-charged scan into monoisotopic neutral mass values. ProSight PC experiment was created generating an XML file in ProSight Upload Format (PUF). From PUF files, searches were performed using the “Absolute Mass and Single Protein” search options against a house database made from sequence of PREDICTED: beta-nerve growth factor [Vicugna pacos] (protein accession number XP_006213759.1). Searches were performed using the following parameters: 2 Da for the average precursor, 10-15 ppm for fragment ions mass tolerance. Proposed sequences with P-score < 1 x 10^-4^ were considered positively identified with a minimum of 10 matching fragment ions with 4 consecutive b or y fragment ions. Interpretation was performed for the two major charge states (8+ and 9+) with a manual, iterative process with variable parameters with or without disulfide bridges) independently tested to maximize the coverage sequence.

*Animals*

Animals were maintained under a 12-hour light, 12-hour dark cycle with food and water *ad libitum*. To obtain dated pregnancies, 1 male was introduced per cage at 5 pm and was removed at 9 am the next day, after three days of litter exchange between male and females. Vaginal plugs were detected the next morning.

1. Labas, V. *et al.* Analysis of epididymal sperm maturation by maldi profiling and top-down mass spectrometry. *J Proteomics* 113, 226–43, DOI: 10.1016/j.jprot.2014.09.031 (2015).
2. Keller, A., Nesvizhskii, A. I., Kolker, E. & Aebersold, R. Empirical statistical model to estimate the accuracy of peptide identifications made by ms/ms and database search. *Anal Chem* 74, 5383–92 (2002).
3. Nesvizhskii, A. I., Keller, A., Kolker, E. & Aebersold, R. A statistical model for identifying proteins by tandem mass spectrometry. *Anal Chem* 75, 4646–58 (2003).
4. Ishima, R. & Torchia, D. A. Error estimation and global fitting in transverse-relaxation dispersion experiments to determine chemical-exchange parameters. *J Biomol NMR* 32, 41–54, DOI: 10.1007/s10858-005-3593-z (2005).
